# Theoretical study on narrow Fano resonance of nanocrescent for the label-free detection of single molecules and single nanoparticles

**DOI:** 10.1039/c7ra12666b

**Published:** 2018-01-19

**Authors:** Chunjie Zheng, Tianqing Jia, Hua Zhao, Yingjie Xia, Shian Zhang, Donghai Feng, Zhenrong Sun

**Affiliations:** State Key Laboratory of Precision Spectroscopy, East China Normal University Shanghai 200062 P. R. China tqjia@phy.ecnu.edu.cn

## Abstract

This paper reports a narrow Fano resonance of 3D nanocrescent and its application in the label-free detection of single molecules. The Fano resonance depends not only on the gap size but also on the height. The Fano resonance originates from the interference between the quadrupolar mode supported by the horizontal crescent and the dipolar mode along the nanotip. When the height of 3D nanocrescent is 30 nm, the width of Fano resonance is as narrow as 10 nm. The narrow linewidth is caused by the strong narrow resonant absorption coming from the dipolar mode of nanotip overlapping with the quadrupolar mode of nanocrescent, where the absorption spectra are calculated under a horizontal incident light. The narrow Fano resonance is highly sensitive to a single nanoparticle trapped by the nanocrescent. The wavelength shift increases linearly with the refractive index with the relation of Δ*λ* = 22.10*n* − 28.80, and increases with the size of trapped nanoparticle following a relation of Δ*λ* = 0.826 × *r*^1.672^. These results indicate that if a protein nanoparticle with radius of 2.5 nm is trapped by the nanocrescent, the shift is as large as 4.03 nm.

## Abstract 2

The narrow Fano resonance caused by the strong narrow resonant absorption is highly sensitive to a trapped nanoparticle.

## Graphical abstract

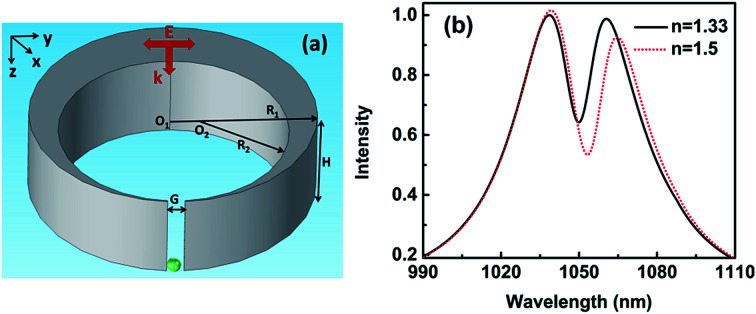

## 1. Introduction

Surface plasmon polaritons (SPPs), with the compact storage of optical energy in electron oscillations concentrated at a metal/dielectric interface, are the key to breaking down the diffraction limit of conventional optics. They have opened up a wide range of applications, such as negative refractive index, lasing, nonlinear optics, especially biosensing.^1–7^ Plasmonic sensors are most widely used based on the concept of surface plasmon resonance (SPR), which is highly sensitive to the change of the refractive index of surrounding media. For example, the resonance frequency shift of a plasmonic nanocavity upon the adsorption or trapping of a single nanoparticle was reported by Zhang *et al.*^8^ In addition, Chen *et al.* studied surface-enhanced Raman scattering *via* a plasmonic nanogap between two Ag nanoparticles, which worked at the single-molecule level.^9^

The interaction between the nanoparticle and the resonant optical modes within the cavity leads to a shift in the resonance frequencies, providing a mechanism for particle sensing. It provides rich and critical molecular information in physical, chemical, and biological sciences.^10–17^ Recently, surface-enhanced Raman scattering,^10^ optical microcavities,^11^ optical trapping in nanoholes,^12^*etc.* were studied for the label-free detection of single molecules (*e.g.*, virus, protein), which allows the object to be detected in the native state in real time.^10–17^ Working at the single-molecule level represents the ultimate practical sensitivity limit. The detection limit of the resonance wavelength shift is of 10^−5^ nm by using a plasmonic–photonic hybrid microcavity to detect single thyroid cancer marker and bovine serum albumin proteins.^14^ Trichet *et al.* presented a new kind of nanotweezers based on open microcavities, which opened the way to the realization of a real-time monitoring system for reducing potential damage to biological samples with a low intracavity power.^18^

Plasmonic Fano resonances arise from the coherent interference of a narrow dark mode and a much broader bright one or a continuum, or from the hybridization between spectrally-overlapped bright modes of nanoparticle clusters, leading to a characteristic asymmetric line shape.^19–24^ Fano resonances are drawing intense interest due to their promising applications in the optical imaging, switching, nonlinear and biochemical sensors.^25–29^ Especially, they have been shown to be much more sensitive to the changes in geometry or local dielectric environment than the primitive modes of the nanostructure because of their microscopic origin as an interference phenomenon, which highly enhances the sensitivity of localized surface plasmon resonance sensors. For example, Cheng *et al.* experimentally demonstrated ultrasensitive detection and characterization of molecules with an asymmetric infrared plasmonic metamaterial on account of a sharp Fano resonance.^25^ In addition, Fano-resonant response of plasmonic metamaterials can be tuned to the vibrational modes of the target biomolecules for ultrasensitive spectroscopy. Wu *et al.* demonstrated the identification of molecular monolayers at the single-molecule level.^26^ However, the significant role of absorption is neglected.^27^ Strong radiative and nonradiative losses of plasmonic nanostructures will result in a broad line width, which can restrict the sensing performance. However, the radiation damping at Fano dip is very low, which gives rise to a very narrow resonance linewidth and is crucial for biosensing.^28,29^

Nanocrescents have attracted a lot of attention because of plasmon coupling and sharp features. Sharp-tip nanostructures have an optical property of lightning rod effect, and can focus the optical energy into nanoscale probe volumes, which makes the optical processes and photochemistry at molecule level.^30,31^ Liu *et al.* presented a systematic theoretical study of tip-enhanced Raman spectroscopy imaging of single molecules to resolve intricate molecule vibrations with atomic resolution.^30^ The potential application in ultrasensitive biomolecular detection is due to the optical properties of polarization intensitive, highly efficient, and tunable light harvesting.^32–36^

Fano resonance is sensitive to the change of dielectric environment and nano-tip is widely used in single molecular Raman scattering. Based on the above two aspects, we designed a 3D nanocrescent for the application in label-free detection of single nanoparticles.^37^ The Fano resonance was attributed to the interference between the quadrupolar mode supported by the horizontal crescent and the quadrupolar mode supported by the nanotip oscillating along the height direction. However, the linewidth of Fano dip was larger than 30 nm, which restricted the sensitivity for single molecular detection.

In this paper, we reports a narrow Fano resonance of 3D nanocrescent and its application in the label-free detection of single molecular. The dependences of the Fano resonance on the height and the size of the gap of nanocrescent are studied, and the Fano resonance is proposed to be originated from the interference between the quadrupolar mode supported by the horizontal crescent and the dipolar mode along the nanotip. When the height of 3D nanocrescent is 30 nm, the width of Fano dip is as narrow as 10 nm. The narrow linewidth is caused by the strong narrow resonant absorption coming from the dipolar mode of nanotip overlapping with the quadrupolar mode of nanocrescent. The sensor based on the Fano resonance of 3D nanocrescent is highly sensitive to a nanoparticle trapped in the nanogap. The influences of the refractive index, size and position of the trapped nanoparticle on the sensitivity are studied, which are much higher than the results of ref. 37 because of the narrower Fano dip.

## 2. Structure model and numerical simulation methods

**Fig. 1:**
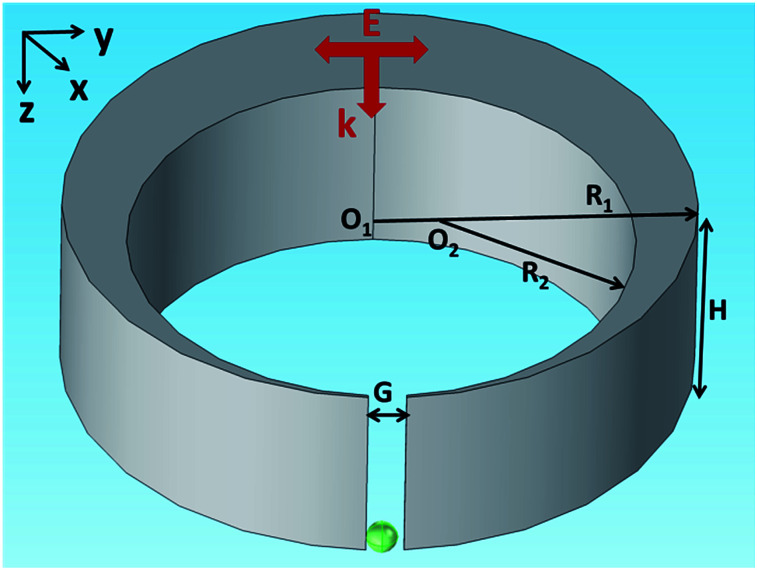
Schematic diagram of the nanocrescent with the vertical incident light. The green sphere indicates the nanoparticle under detection.

The nanocrescent structure is shown in Fig. 1, where height is *H* = 30 nm, outer radius is *R*_1_ = 60 nm, inner radius is *R*_2_ = 40 nm, and the center distance is *O*_1_–*O*_2_ = 19 nm. The tip-to-tip gap is *G* = 6 nm and the wall width of the tip is 1 nm. The nanocrescent is made of silver with the permeability *μ* = 1 and the complex permittivity coming from ref. 38. The 3D nanocrescent is placed on a glass substrate with a refractive index of 1.5, and immersed in an aqueous environment (*n*_w_ = 1.33). The electric field *E* of a vertical incident light (wave vector *k*//*z* axis) irradiated down to the nanocrescent is parallel to the *y* axis (*E*//*y*), as shown in Fig. 1.

The optical properties of the nanocrescent are studied by finite element method (COMSOL) adopting adaptive mesh. A spherical perfectly matched layer is used to absorb all outgoing radiation and to eliminate the reflections at the domain boundaries. During the numerical simulation, the maximum element size of local adaptive mesh refinement is 20 nm and the minimum is 0.2 nm.^32^

## 3. Results and discussion

### 3.1 Fano resonance

#### 3.1.1 Narrow Fano resonance

**Fig. 2:**
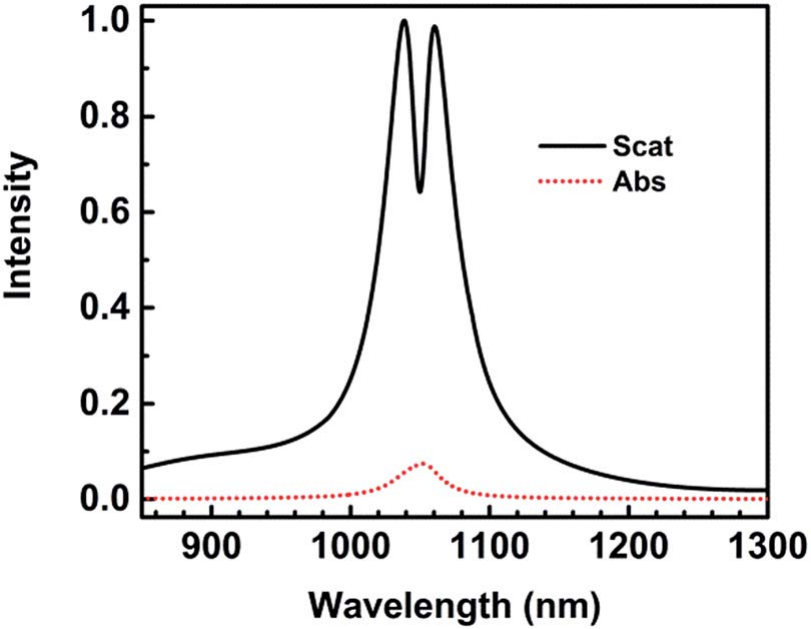
The scattering and absorption spectra of nanocrescent for *H* = 30 nm.

The scattering and absorption spectra of nanocrescent are shown in Fig. 2. The scattering spectrum shows a deep and narrow Fano resonance at the wavelength of 1050 nm with a characteristic asymmetric line shape. The parameters of the geometric structure of the 3D nanocrescent are same as those shown in Fig. 1. Fano resonance was proposed to be induced by the interference between bright superradiant and dark subradiant modes.^21,22^ Radiative losses at the Fano dip were effectively suppressed due to excitation of the dark subradiant modes, which was evaluated by the electric far field.^28^ The far field of the radiation originated from the nanogap at the blue peak (1038 nm), Fano dip (1050 nm) and red peak (1060 nm) are calculated. The results show that the radiation at the Fano dip is only of 80% compared with the two peaks, which is decreased by 48% if Fano resonance is absent.

What is to our surprise is that the full width at half maximum (FWHM) of the Fano dip is very narrow, only 10 nm. Many references reported Fano resonances with a width of several tens of nanometers, which depends mainly on dark mode or radiative loss and the coupling effects.^20,21,28,29,39^

#### 3.1.2 Size dependence of Fano resonance

**Fig. 3:**
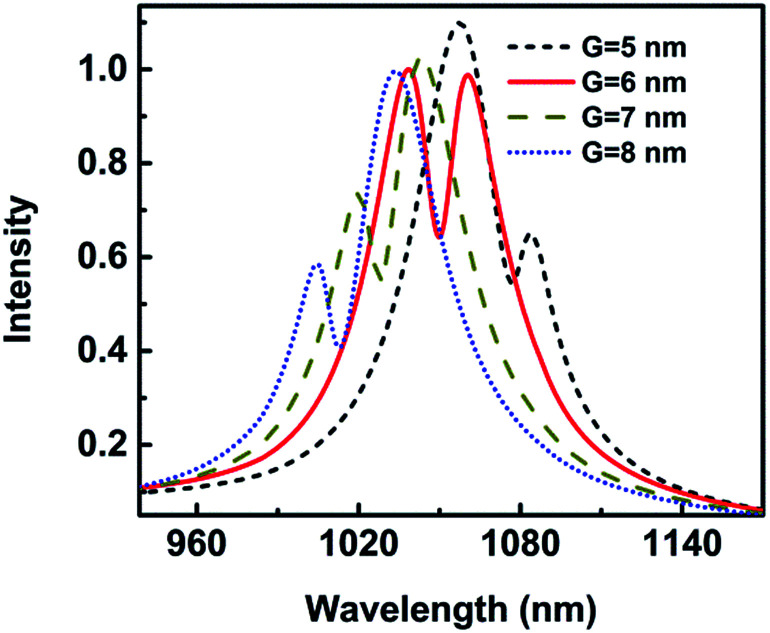
The scattering spectra as a function of the gap size *G* with height *H* = 30 nm.

In order to study the size-dependence of geometrical structures on the Fano resonance, the gap width *G* is varied. At the meantime, the separation *O*_1_–*O*_2_ changes accordingly to make sure the wall width of tip unchanged, while other parameters keep constant. The normalized scattering spectra for *G* = 5 nm, 6 nm, 7 nm, and 8 nm are shown in Fig. 3. It is seen that for *G* = 5 nm the spectrum has an asymmetric broad shape with the Fano dip of 1077 nm. When the gap increases to 6 nm, it leads to a narrow line shape with a Fano dip at the wavelength of 1050 nm. As the gap further increases to 8 nm, the Fano dip blue shifts to 1013 nm. This can be explained that the gap capacitance of a spilt ring is inversely proportional to the gap size *G* (*C* = *εS*/*G*, *ε* is the dielectric constant of the medium filling the gap and *S* is the area of the two plates), and the resonance frequency is inversely proportional to the square root of the gap capacitance.^39,40^ Therefore, as increasing the gap size *G*, the Fano resonance is blue tuned correspondingly. Meanwhile, the wall thickness of crescent increases with larger *G*, which also decreases the coupling of nanotip.^33,35,39^ These two factors make the Fano dips blue shift. Both the Fano spectra shape and resonance wavelength change greatly with the gap between two nanotips. Similar Fano spectra were observed at the scattering spectra of nanocrescent and cut-disk.^41–43^ The scattering spectrum with a double-peak Fano shape was supported by a cut-disk particle due to the interference between two horizontal modes, the bright dipolar mode of the slit disk and the dark quadrupolar modes of the narrow split gap.^42^

In addition, Wu *et al.* found that two distinct peaks in the plasmon band began to emerge with a proper thickness of the crescent-shaped nanohole antenna, which stressed that the resonance was not purely a two-dimensional effect.^41^ Therefore, it is necessary to study the influence of height on the Fano resonance.

**Fig. 4:**
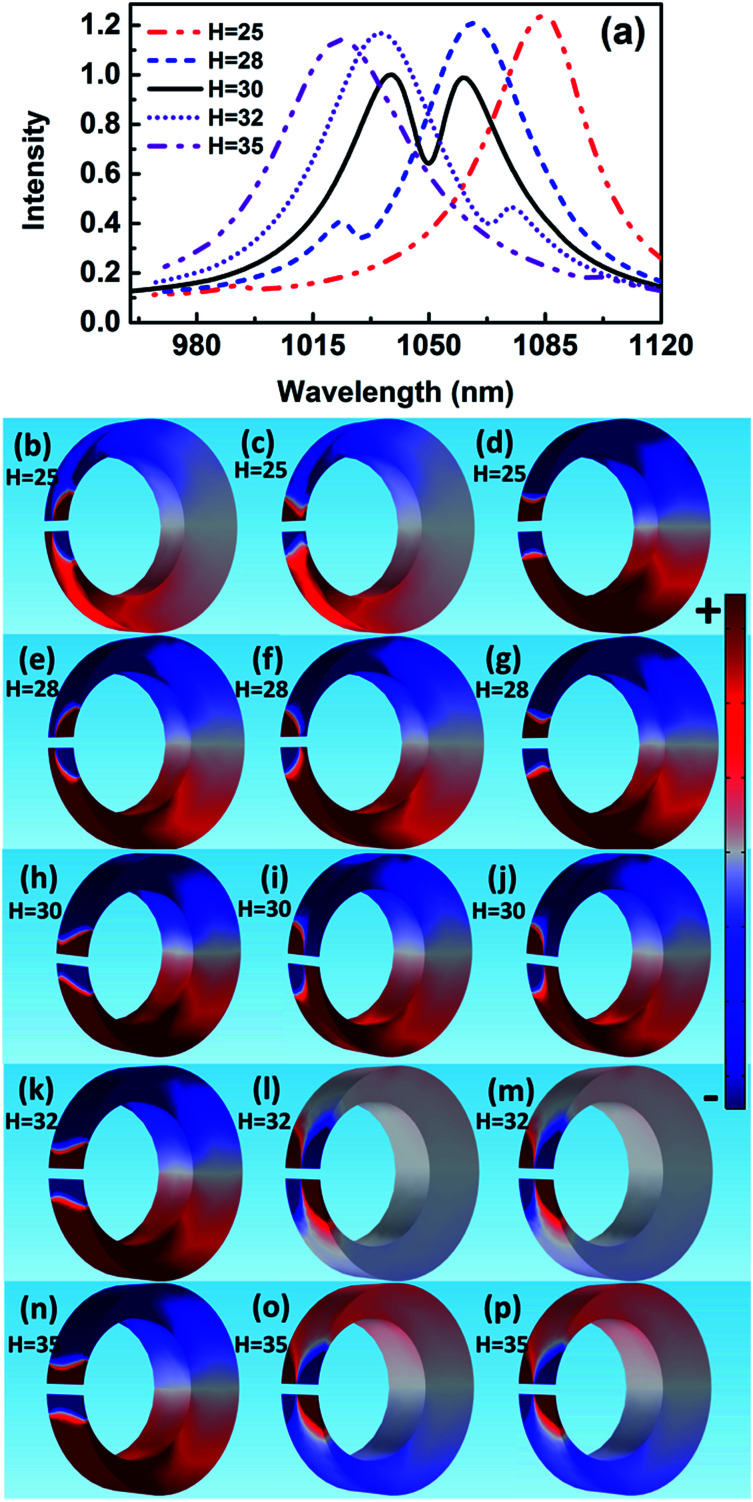
The scattering spectra (a) and charge distribution for height *H* = 25 nm, 28 nm, 30 nm, 32 nm, and 35 nm. The charge distributions at the blue peak (b), Fano dip (c) and red peak (d) for *H* = 25 nm; the blue peak (e), Fano dip (f), and red peak (g) for *H* = 28 nm. The blue peak (h), Fano dip (i), and red peak (j) for *H* = 30 nm. The blue peak (k), Fano dip (l), and red peak (m) for *H* = 32 nm. The blue peak (n), Fano dip (o), and red peak (p) for *H* = 35 nm.

The scattering spectra and charge distributions are studied by changing the height of nanocrescent from 25 to 35 nm, as shown in Fig. 4. For height *H* = 25 nm, the scattering spectrum exhibits a nearly uncoupled plasmonic modes, shown in Fig. 4(a). The main peak appears at 1084 nm, and a very weak peak at 991 nm with little spectral overlap. The charge distribution shows a quadrupolar mode except for a little hybridization shown in Fig. 4(b–d). When the height increases to *H* = 28 nm, the Fano dip shifts to 1030 nm and FWHM is 33 nm. The charge distribution shows a vertical dipolar mode along the nanotip shown in Fig. 4(f). When the height increases to *H* = 30 nm, the Fano resonance is perfectly excited. The charge distributions are shown in Fig. 4(i). Similar to the height *H* = 25 nm, the plasmonic modes nearly uncoupled each other for height *H* = 35 nm. With the height increasing, the Fano dip red shifts and the charge distribution shows a clearly vertical dipolar mode along the nanotip shown in Fig. 4(f, i, l and o). In a word, the peak of the quadrupolar mode is blue-shifted with the height increasing because of the dispersion relation of the nanostructure plasmon, and the gradual red shift of Fano dip is mainly caused by the increase in the capacitance of the spilt gap.^39,40^

In addition, there is only a quadrupolar mode supported by nanocrescent when the height *H* = 20 nm and no Fano interference is present. The 2D nanocrescent was calculated. The quadrupolar mode was a single peak without any spilt because of no vertical charge oscillation along the nanotip.^32^

These results indicate that Fano resonance is observed for appropriate height. The charge distributions and the spectra demonstrate that the greater the thickness is, the longer the resonance wavelength is. The origin of the Fano resonance is due to the interference between a horizontal quadrupolar mode supported by the crescent and the vertical dipolar mode along the nanotip.

The spectral features associated with the charge distributions provide a clear picture regarding the origin of the Fano resonance, which can be explained that the quadrupolar resonance mode of the nanocrescent is effectively excited by normal incident light. It further excites resonantly the vertical dark mode. The vertical dipolar mode interferes strongly with the horizontal quadrupolar resonance mode of the nanocrescent, forming the narrow deep Fano resonance. Recently, Pellarin *et al.* reported Fano resonance of nanocube dimers, which is caused by the destructive interference in the strong coupling between a highly localized modes and a highly radiating longitudinal dipolar plasmon of the dimer.^20^ In addition, Liu *et al.* studied Fano resonances of 3D plasmonic structures formed by integrating vertical U-shape spilt-ring resonators along a planar metallic hole array. The surface plasmon polaritons of the metallic hole array excited by normal incident light can efficiently activate vertical electric currents (dark mode) in the vertical spilt-ring resonators. The vertical spilt-ring resonators interfered with the metallic hole array, and induced the deep Fano resonance.^43^ Similarly, the Fano resonance of the 3D nanocrescent is originated from the strong interference between the horizontal quadrupolar mode of the nanocrescent and the vertical dipolar mode along the nanotip.

#### 3.1.3 Polarization dependence of Fano resonance

**Fig. 5:**
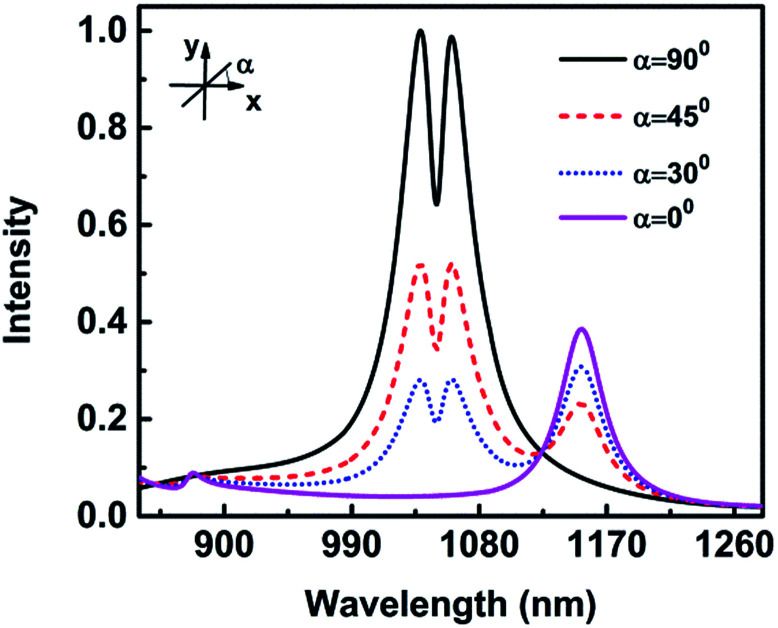
The scattering spectra at normal incidence illumination with different polarization angles.

The size dependences indicate that the Fano resonance is due to the interference between the vertical dipolar mode along the nanotip and a horizontal quadrupolar mode supported by the crescent. To better support this mechanism, the dependence of Fano resonances on the incident light polarization is performed. Fig. 5 shows the scattering spectra for normal incident light, while the polarization angle *α* changes from 0° to 90°. Here *α* = 0° (90°) corresponds to *E*//*x*(*y*) direction. For *α* = 0°, the scattering spectra display only a strong dipolar mode at 1152 nm and a weak peak at 878 nm. No Fano resonance is observed. As *α* increases to 30°, a new Fano-shape resonance at 1049 nm emerges between the two scattering peaks, which is due to the *y*-component of electric field. The Fano-shape resonance becomes stronger as *α* increases to 45°. A deep, narrow and strongest Fano resonance appears for *α* = 90°. These results display that the Fano-shape resonance only can be excited by the *y*-component of the electric field and the *x*-component cannot, which is determined by the surface plasmon modes.^44^ These results further support the mechanism that Fano resonance is due to the interference between the vertical dipolar mode along the nanotip and a horizontal quadrupolar mode supported by the nanocrescent.

### 3.2 The dark mode

#### 3.2.1 The narrow absorption spectra

**Fig. 6:**
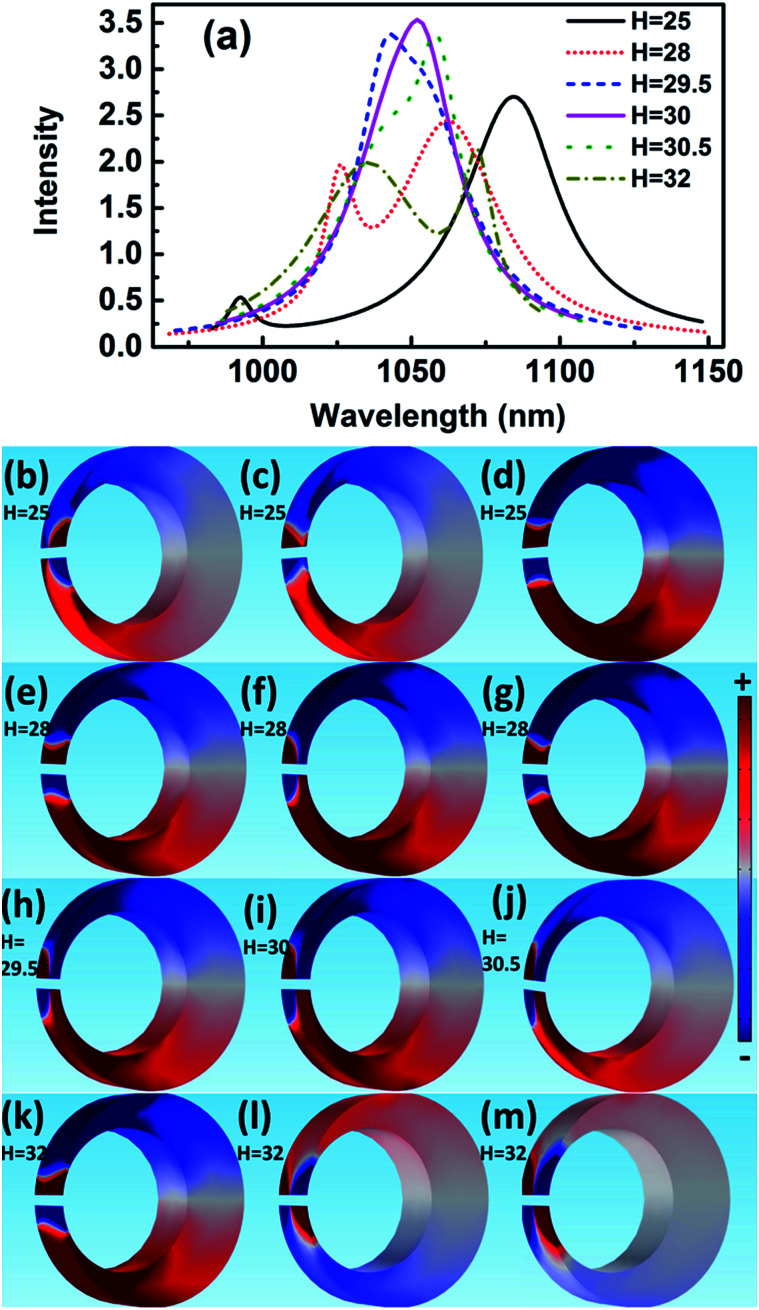
The absorption spectra (a) and charge distribution for height *H* = 25 nm, 28 nm, 30 nm, 32 nm, and 35 nm. The charge distributions at the blue peak (b), Fano dip (c) and red peak (d) for *H* = 25 nm; the blue peak (e), Fano dip (f), and red peak (g) for *H* = 28 nm. The Fano dip (h–j) for *H* = 29.5 nm, *H* = 30 nm. *H* = 30.5 nm, respectively. The blue peak (k), Fano dip (l), and red peak (m) for *H* = 32 nm.

In order to explain the mechanism of Fano resonance, the direction of incident light is changed along with *x* direction and the electric field *E* along with *y* direction, which can excite the dark mode in the vertical direction. The absorption spectra and charge distributions for the height in the range of 25 to 32 nm are shown in Fig. 6. For height *H* = 25 nm, the absorption spectrum has two peaks at 992 and 1084 nm. The charge distribution indicates that peak at 1084 nm is a quadrupolar mode and the other shows hybrid mode in the vertical direction shown in Fig. 6(a–d). The spectra are less overlap and the modes are weak coupling. When the height increases to 28 nm, the absorption spectrum is spilt, indicating the distinctly asymmetric Fano resonance. The charge distribution shows that the charge oscillates up and down to form dipolar mode in the vertical direction at the Fano dip, and only slightly hybrid modes happen at two peaks shown in Fig. 6(e–g), which indicates that the Fano resonance is formed by the interference of dipolar mode in the vertical direction and quadrupolar mode in the horizontal direction. For height *H* = 30 nm, the absorption spectrum is only a narrow asymmetric resonance peak with the FWHM = 37 nm, which corresponds to the scattering dip at the same wavelength, and the charge shows a clearly quadrupolar mode in the horizontal direction and dipolar mode in the vertical direction in Fig. 6(i). The narrow strong absorption peak is thought to be generated by spectra superposition of two modes, caused by resonance.

When the height is 30.5 nm, the spectrum with the FWHM = 39 nm is hybridization because of strong coupling. When the height increases to 32 nm, the absorption spectrum shows a Fano-shape again. Especially, it is only quadrupolar mode at short or long wavelength when it is far away from Fano resonance for every height, which indicates again the Fano-shape is generated by the coupling. When the height is 60 nm, it is two quadrupolar modes that interfere with each other to generate Fano resonance, but the height supports high-order mode and complex hybridization, causing a wide linewidth.^37^ As a result, the Fano resonance is highly depended on the height. The greater the height is, the longer the resonance wavelength is. In the end, Fano resonance is observed for appropriate height when other parameters constant, which indicates that the origin of the Fano resonance is due to the interference between the vertical dipolar mode along the nanotip and the horizontal quadrupolar mode supported by the crescent.

The peak of the absorption spectra for *H* = 30 nm is generated by the superposition of the two modes, which generates a narrow linewidth by strong resonance. In addition, the intensity of the absorption spectrum for height *H* = 30 nm is the most strong normalized by the incident light, which indicates that the optical absorption efficiency is at a high level.

#### 3.2.2 The origin of narrow linewidth

To better understand the strong narrow linewidth, we introduce a simple mathematical mode to fit the Fano lineshape as following.^19,29^

##### 3.2.2.(i) A mathematical mode for Fano resonance

The resonance in the entire system is given by the product of asymmetric and symmetric resonances.

1 *F*_total_ = *F*_a_*F*_s_

The Fano-like resonance with an asymmetric shape is functioned as:2
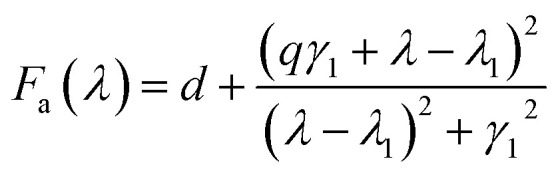


The symmetric Lorentzian lineshape is function as:3
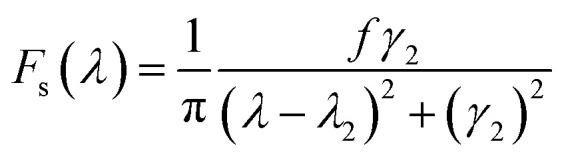
where *λ*_*i*_ and *γ*_*i*_ (*i* = 1, 2) are the central wavelength and a half of the linewidth of the resonance, respectively. *q* is Fano parameter describing the degree of asymmetry, and *d* and *f* are constant factors describing the background and the overall peak height, respectively. The 3D nanocrescent is used as like-atom system for simplifying calculation. The coupling between asymmetric shape and symmetric shape gives rise to the absorption spectra.

**Fig. 7:**
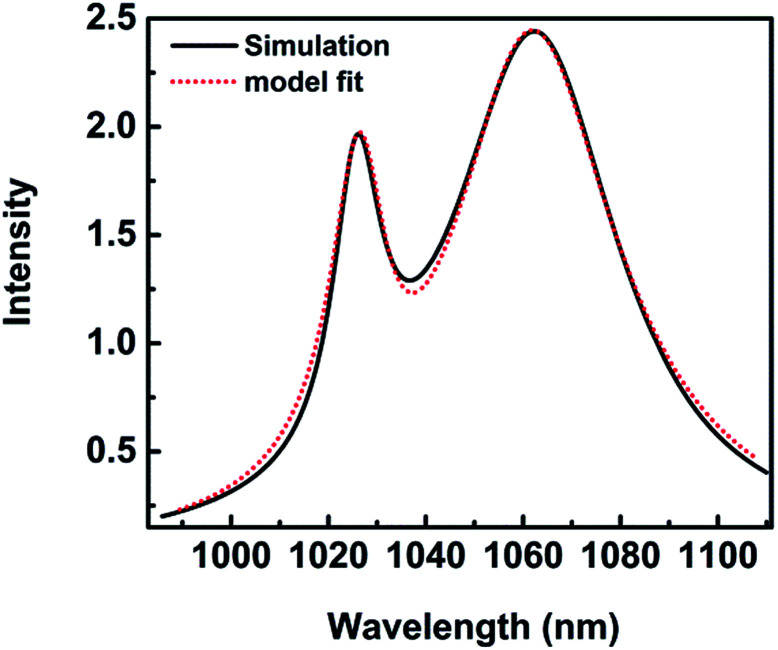
The spectra for height *H* = 28 nm. (Black solid) numerical simulations; (red dot) fit with eqn (1)*F*_total_.

The spectra coming from numerical simulation and fit with mathematical formula for height *H* = 28 nm are shown in Fig. 7. The fit result makes an agreement with the numerical simulation, which indicates the reasonability of the mode. *λ*_1_ = 1026.75 nm is the position of an asymmetric, narrow line and *λ*_2_ = 1061.64 nm is the position of a symmetric, broad one. A dip emerges in the spectrum at wavelength of 1037.83 nm, resulting in a Fano lineshape. The Fano profile is a consequence of the interaction between a narrow mode and a wide mode from the detailed fit with eqn (1). *q* = 3.85 describes the degree of asymmetry.

**Fig. 8:**
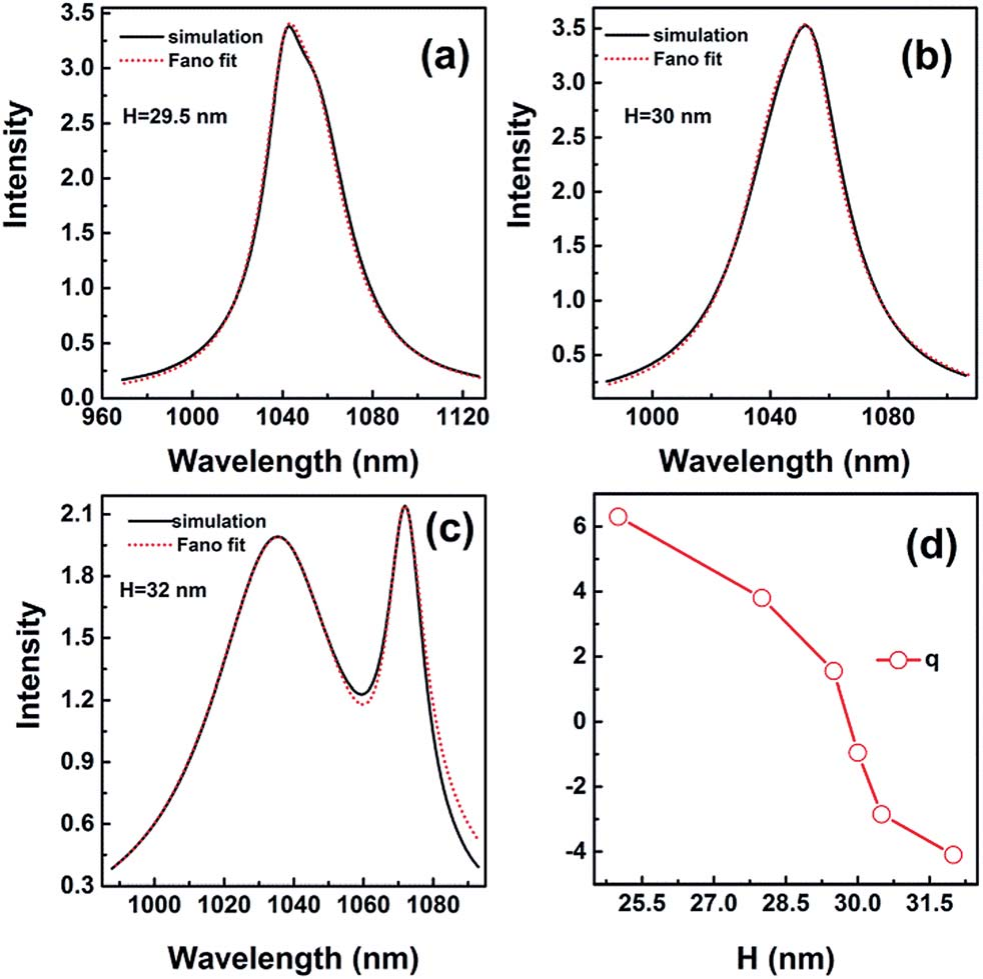
The spectra for height *H* = 29.5 nm (a), *H* = 30 nm (b), and *H* = 32 nm (c). (d) *q* as a function of height. (a–c) (Black solid) numerical simulations; (red dot) fit with eqn (1)*F*_total_.

The function fitting to the Fano profile with different heights is shown in Fig. 8. The Fano profile takes the form of a pseudo-Lorentzian when the height is 30 nm with *q* = −0.95 shown in Fig. 8(b). The value of *λ*_1_ is 1050.85 nm and *λ*_2_ is 1046.41 nm. When the height is *H* = 32 nm, the value of *λ*_1_ is 1070.81 nm and *λ*_2_ is 1035.92 nm. The lineshape for *q* = −4.1 distinctly shows strong asymmetry shown in Fig. 8(c). Compared with the spectra for height *H* = 30 nm, the absorption spectrum becomes wider because of complicate hybridization for height *H* = 29.5 nm. The value of *λ*_1_ is 1042.46 nm and *λ*_2_ is 1053.08 nm, and *q* is 1.55 shown in Fig. 8(a). As the height is 30 nm, the value of *λ*_1_ is in close proximity to *λ*_2_, which indicates the strong resonance occurring when the asymmetric narrow mode nearly overlaps with the symmetric broad one.

The sign of *q* determines the fast rise of the line shape is on the low or high-energy side of the central wavelength of bright mode. *q* varies in the range of −4.1 to 6.3 with height increasing shown in Fig. 8(d). The absolute value of *q* decreases first and then increases, *i.e.* the asymmetry of resonance becomes weak and strong again, as shown in Fig. 8(a–c). When the height is *H* = 32 nm, absolute value of *q* is larger than 4.0, the lineshape shows distinctly asymmetry. For small absolute values of *q* (*q* = −0.95), the Fano profile takes nearly the form of a symmetric lineshape when the height is 30 nm. However, the value of *q* is not equal to 0, which indicates the lineshape is somewhat asymmetric being the result of coupling.

As a result, a simple mathematical mode indicates the Fano resonance arises from the interference of an asymmetric narrow resonance and a symmetric broad resonance. The line width of the resonance is mainly decided by the modes coupling. Importantly, the narrow linewidth of the absorption spectra is caused by strong resonance because of the two modes nearly overlapping when the height is *H* = 30 nm.

##### 3.2.2.(ii) The reason of narrow linewidth of absorption spectra

**Fig. 9:**
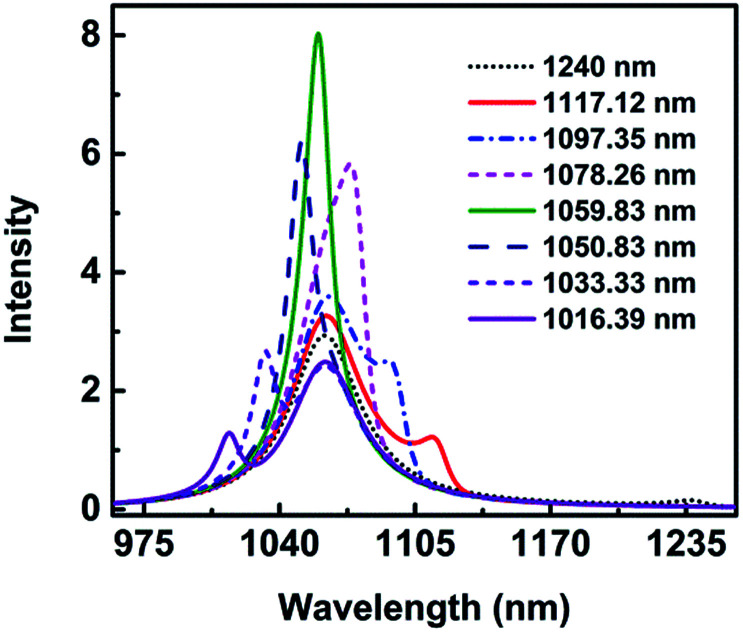
The spectra as a function of *λ*_1_.

In order to better comprehend the narrow linewidth of absorption spectra, the wavelength *λ*_1_ is changed with other parameters constant as mentioned above for height *H* = 28 nm. The wavelength *λ*_1_ of the asymmetric narrow line changes is changed from 1240.0 nm to 1016.39 nm, while *λ*_2_ keeps at 1061.64 nm. The spectra are shown in Fig. 9. When *λ*_1_ is at 1240.0 nm, the main peak appears at 1061.0 nm and a very weak peak at 1237.23 nm, which indicates the two modes are less coupling. As *λ*_1_ decreases to 1117.12 nm, a shallow dip appears. Surprisingly, it is only a peak when *λ*_1_ is in the range of 1078.26 nm to 1050.83 nm. The spectra are clearly hybridized at the wavelength of 1050.83 nm and 1078.26 nm with the FWHM being 32 nm and 20 nm, respectively. As *λ*_1_ moves to 1059.83 nm, very close to *λ*_2_ = 1061.64 nm, the absorption peak is the most strong, and the FWHM is the most narrow, only 17 nm. These results indicate the two modes overlap well and strong resonant absorption happens.

By fitting the analytical formula to the numerical simulations, the line shape of Fano resonance matches well with the spectra shown in Fig. 6(a). However, the intensity of the resonance absorption spectrum is much higher and the FWHM is far narrower than those of numerical simulation results. The reason is that only the interference is taken into consideration in eqn (1)–(3), while other factors, such as the hybridization caused by the height, the asymmetry parameter, and so on are neglected. In consequence, the reason of the narrow linewidth for the absorption spectra at the height *H* = 30 nm can be concluded that the spectra between the dipolar mode in the vertical direction and quadrupolar mode in the horizontal direction overlap and the strong resonance occurs.

## 4. The application of nanocrescent in plasmonic sensing

### 4.1 The detection of nanoparticle

Nanocrescent is a typical plasmonics, which is widely used in ultrasensitive biomolecular detection, especially for single-molecule detection.^32–36^ Fano resonance structure becomes more and more interesting for detecting single molecule.^25,26^ Furthermore, narrow Fano dip is demanded to achieve low limit of detection in the biochemical sensing platforms. In the following, we will study the detection of single nanoparticles trapped in the nanogap by using the Fano resonance.

The detection of protein molecules or virus based on the coupling of surface plasmon polaritons in metallic nanostructure offers significant opportunity in the field of medical research and clinical diagnostics.^10,12,14–17^ In our model, the green sphere indicates the protein under detection as shown in Fig. 1. The refractive index of protein nanoparticle is set as 1.5 for different types of proteins changing slightly in the range of 1.45–1.5 and the radius is set as 2.5 nm to fit the 3–8 nm thick monolayer of protein.^13,26,45–47^ In order to avoid computation errors due to re-meshing the geometry, we simply set the refraction index of the nanoparticle domain to be that of water in the case of no nanoparticle.

**Fig. 10:**
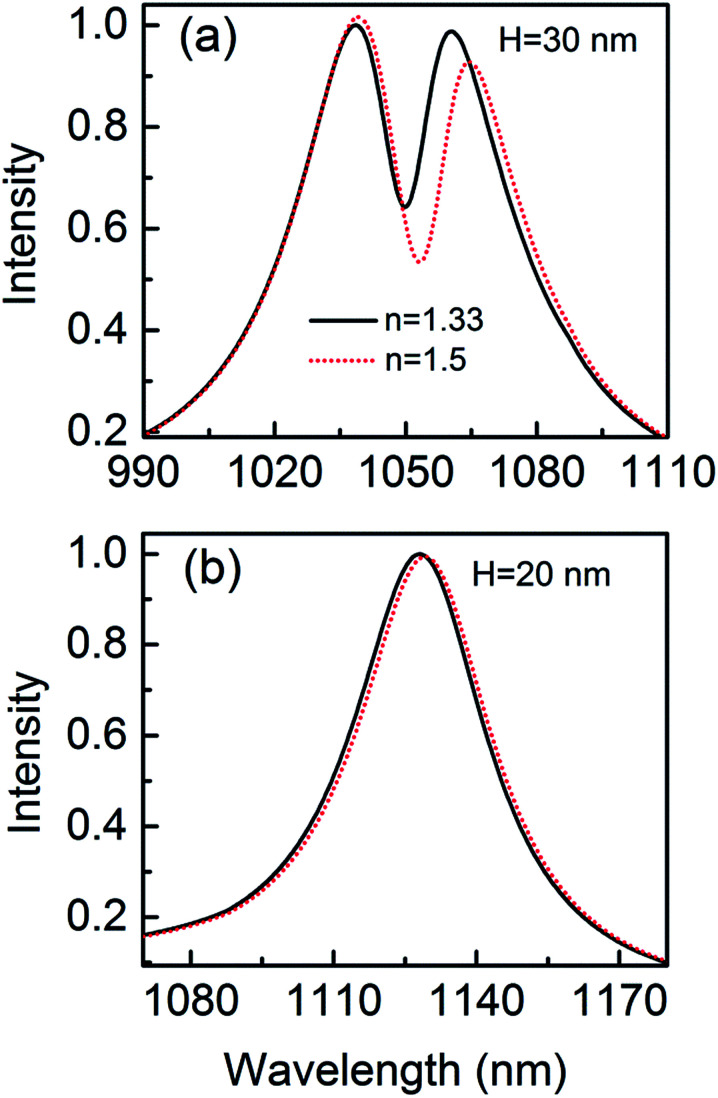
The scattering spectra with nanoparticle (*r* = 2.5 nm) trapped by the nanocrescent for *H* = 30 nm (a) and *H* = 20 nm (b).

The narrow Fano dip has a great influence on the sensitivity. When a protein is trapped in the gap, Fig. 10 shows that there is a resonance shift of 4.03 nm that is 5 orders of magnitude higher than the detection limit.^14^ The resonance shift is calculated at the long wavelength side of the Fano dip. The spectral shifts can be more easily distinguished due to the sharper resonance. The parameters of the geometric structure of the 3D nanocrescent are same as those shown in Fig. 1. For the nanocrescent with height *H* = 60 nm, the dark mode is the quadrupolar mode supported by the nanotip oscillating along the height direction.^37^ The wavelength shift is 1.86 nm when the nanoparticle (*r* = 2.5 nm) is trapped in the nanogap, which is less than a half of the nanocrescent with *H* = 30 nm. Compared with the spilt ring with the same size except for the sharp tip, the sensitivity of the 3D nanocrescent is improved by several times of magnitude due to the narrow linewidth at the Fano dip, which indicates that the Fano resonance is very sensitive to a trapped nanoparticle and holds promise for the ultrahigh sensitivity.

In addition, in order to interpret the sensitivity dependence on the Fano resonance, the phenomenon of no Fano resonance is also studied. When the height is 20 nm, the Fano resonance disappears because the height cannot support vertical dipolar mode, as shown in Fig. 10(b). The wavelength shift is only 1.08 nm when the nanoparticle is trapped by the nanocrescent, and it is only a quarter by using Fano resonance of 3D nanocrescent.

**Fig. 11:**
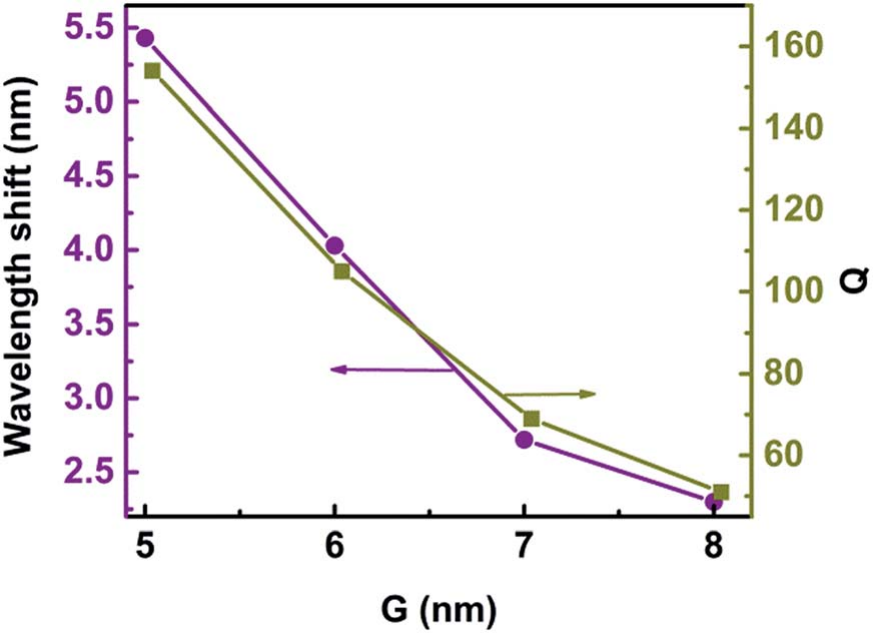
The wavelength shift and *Q* as a function of the gap width *G*.

Label-free single-molecule detection based on optical trapping has great potential in microbiology applications because of its ability to trap tiny bioparticles without inducing damage.^8–15^ Trichet *et al.* presented a new kind of nanotweezers based on open-access microcavities with the abilities of situ detection, trapping, and measurement of the polarizability along with the trap strength, which opens the way to the realization of a real-time monitoring system for single nanoparticle properties.^18^ In our model, the 3D nanocrescent is also an open microcavity. The quality factor (*Q*) is defined as *Q* = *λ*/Δ*λ*, where *λ* is the central wavelength of the Fano resonance and Δ*λ* is the difference between the scattering peak and dip.^48^ For *H* = 30 nm and *G* = 6 nm, *Q* is as high as 105 because of the suppressed radiative loss of the Fano resonance, as shown in Fig. 11. The spilt of nanocrescent provides a confined optical mode and nanoparticles can directly diffuse to the region with the highest electromagnetic field, which can determine the optical response to an electromagnetic field. In one word, the sensor by using a Fano resonance of 3D nanocrescent is high sensitivity caused by a narrow linewidth and can provide a real-time detection system for single nanoparticles in the future.

### 4.2 The sensitivity of 3D nanocrescent

The metallic tip, which can resonate with the local mode of the surface plasmon, can provide high-sensitive and high-spatial-resolution optical analytical approach, which would help its application for single-molecule investigations into intramolecular coupling, structure, and vibrational dynamics.^30,31^ The gap width has an influence on the Fano resonance. Fig. 11 shows the wavelength shift and *Q* as a function of the gap width when a protein with radius of 2.5 nm is trapped. The wavelength shift is 2.30 nm when the gap is 8 nm. With the gap decreasing, the wavelength shift increases. The wavelength shift increases to 5.43 nm when the gap is 5 nm. The resonance wavelength shift becomes larger with gap decreasing. This can be explained that the coupling of spilt reduces with the increasing of *G*, causing the weak coupling between the resonance modes of the nanocrescent and nanotips.^33,35^

With the gap increasing, *Q* is reduced, similar to the wavelength shift, which is because the Fano dip becomes wider, as shown in Fig. 3. The sensitivity is highly dependent on *Q*. The larger *Q* is, the higher the sensitivity is. The reason is that the radiation loss of Fano resonance is more suppressed and the electric field is greater enhanced in the narrower gap.

To study the detection limit, the size and shape of nanostructure have been optimized. The nanostructure with height 30 nm and gap 6 nm is chosen due to the Fano profile and stable sensitivity. In the following, we will study the detection of different analytes.

**Fig. 12:**
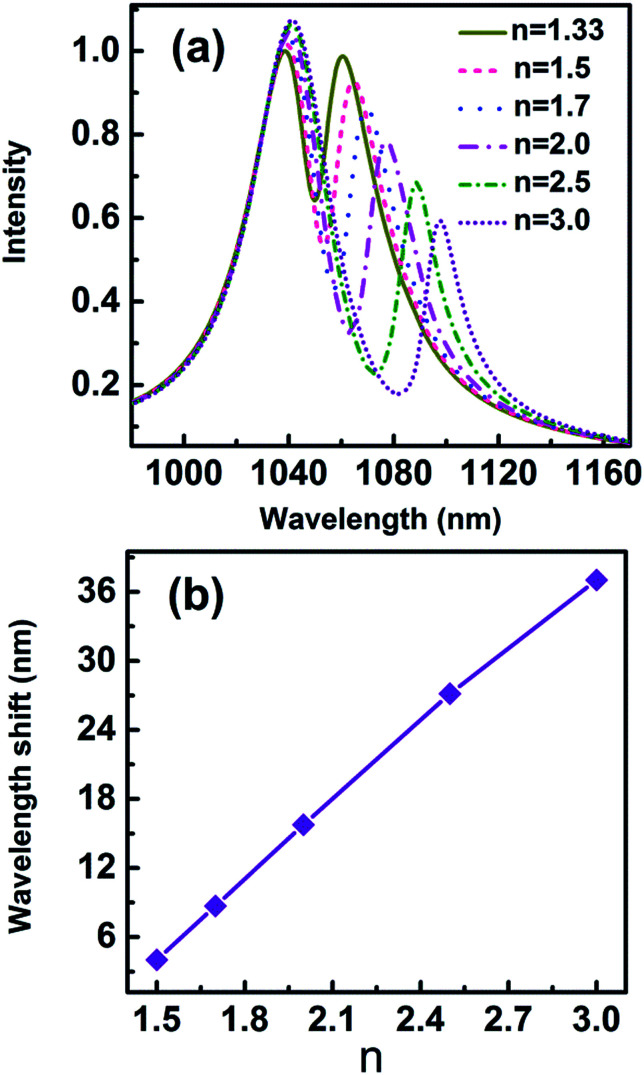
(a) The scattering spectra with different refractive indices of nanoparticles, (b) the wavelength shift as a function of refractive index *n*.

Fano-resonant metamaterials are highly sensitive to changes in geometric parameters and the refractive indices of the surrounding medium. Recently, the molecule detection based on an asymmetric Fano profile has drawn more and more attentions.^25,26^ Cheng *et al.* reported ultrasensitive detection and characterization of polymer molecules based on a sharp, non-radiative Fano resonance caused by an asymmetric infrared plasmonics metamaterial.^25^ Apart from biological molecules, the detections of polymer or semiconductor nanoparticles with refractive index in the range of 1.7–3 were widely studied.^8,9,37^Fig. 12(a) displays the scattering spectra for nanoparticle (*r* = 2.5 nm) with different refractive indices trapped in the gap. The parameters of the geometric structure of the 3D nanocrescent are same as those shown in Fig. 1. The scattering spectra are red shift with refractive index increasing. The wavelength shift is 8.70 nm, 15.77 nm, 27.14 nm, and 37.04 nm when the refractive index of nanoparticle is 1.7, 2.0, 2.5, and 3.0, respectively. The wavelength shift increases linearly with the refractive index and the relation is Δ*λ* = 22.10*n* − 28.80.

Figure of merit (FOM) is used to evaluate the overall performance of sensors, which is defined as *S*/FWHM. *S* = δ*λ*/δ*n* is the refractive index sensitivity equalled to spectral shift per refractive index unit (RIU).^6^ In Fig. 12(b), the sensitivity *S* = 22.12 nm RIU^−1^ and FOM = 2.2 for single nanoparticle with a radius of 2.5 nm are evaluated.

In order to further compare the ability of detection of 3D nanocrescent, the sensor characteristics of crescent without Fano resonance are estimated.^49^ The sensitivity is 0.288 nm RIU^−1^ for a single nanoparticle with radius of 2.5 nm trapped, which is nearly 2 orders of magnitude less than the sensitivity of 3D nanocrescent we designed. And FOM is 0.00169, which is more than 3 orders less. The sensor of two Ag nanoparticles with a 2 nm gap was studied theoretically.^9^ The sensitivity and FOM are 2.05 nm RIU^−1^ and 0.026, less than those of the 3D nanocrescent by 1 order and 2 orders of magnitude, respectively.

**Fig. 13:**
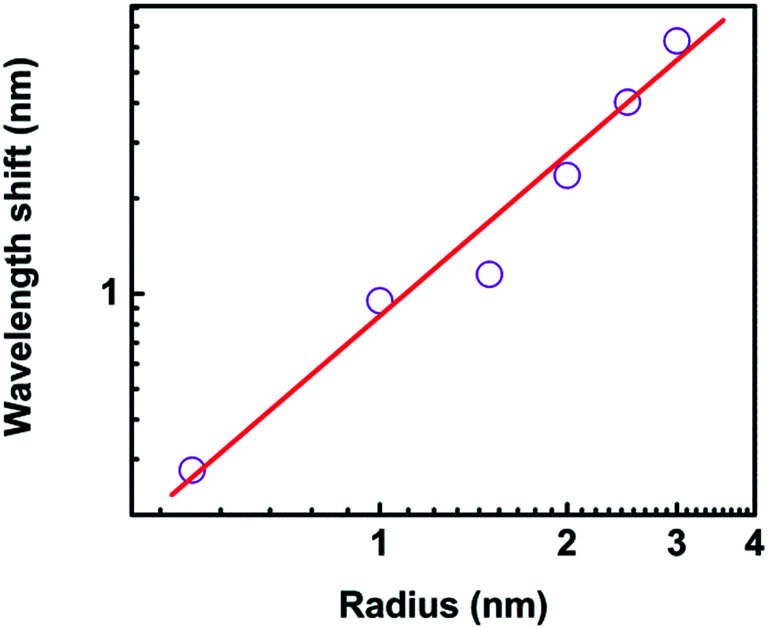
The wavelength shift as a function of the radius of nanoparticle.

The sensitivity dependence on the size of nanoparticle varied from 0.3 nm to 2.5 nm provides an opportunity to investigate the ultimate detection limit, as shown in Fig. 13. The wavelength shift is 0.277 nm, 0.951 nm, 1.141 nm, 2.364 nm, 4.027 nm, and 6.290 nm when the radius of protein is 0.5 nm, 1.0 nm, 1.5 nm, 2.0 nm, 2.5 nm, and 3.0 nm, respectively. The linear fitting of the logarithmic dependences shows a relation of Δ*λ* = 0.826 × *r*^1.672^. It is predicted the wavelength shift is 0.018 nm when the radius is only 0.1 nm, which is still much larger than the detection limit of biosensor platform.^14^ In our model, the quantum effect in the plasmonic system is out of our scope.

It is interesting that the wavelength shift of the nanocrescent is highly dependent on the size of trapped nanoparticle. The reason is that with the size increasing, the effective permittivity between the nanotips is changed, and the effective capacitance changes accordingly, which further results in the increasing of nanocrescent sensitivity.^37^ Zijlstra *et al.* reported the optical detection of single molecules using the surface plasmon resonance of a gold nanorod. They found that the wavelength shift per molecule increased with the molecular weight.^47^ Li *et al.* studied the Fano interference-induced force owing to the phase sensitivity of the interference between adjacent plasmon modes in the particle.^50^ They found the Fano scattering force is ultrasensitive to the particle size, and showed the possibility of size selection of plasmonic nanoparticles with an accuracy of 10 nm.

**Fig. 14:**
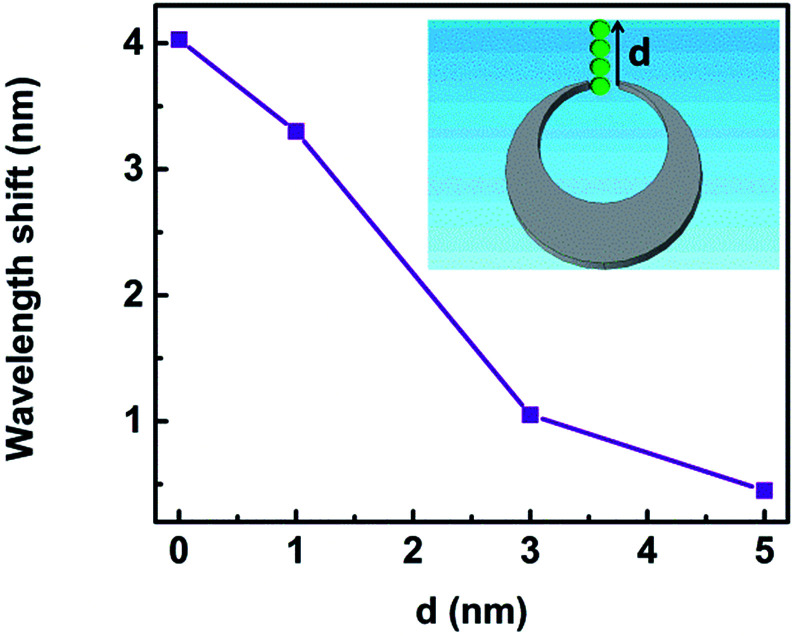
The wavelength shift as a function of the distance *d*.

The resonance shift upon the changes of local environment around the trapped nanoparticle provided guidelines for analyzing and further developing efficient active plasmonic devices.^6,9,51^ We study the wavelength shift of Fano resonance when the nanoparticle is trapped at different locations *d* as sketched in the set in Fig. 14. When *d* changes from 0 to 1 nm, 3 nm and 5 nm, the spectra shift by 4.03 nm, 3.30 nm, 1.05 nm, and 0.45 nm, respectively. The Fano dips become shallower accordingly. The reason is that the field enhancement is largest for *d* = 0 nm, and it decreases rapidly with larger *d*.^6,9,51^ Therefore, the location of the trapped nanoparticle influences the sensitivity of the 3D nanocrescent.

In one word, the high sensitivity and figure of merit mainly take advantages of the strong narrow resonant absorption of dark mode, which further causes a very narrow Fano dip. These are very useful for realizing high-resolution plasmonic biosensors and achieving low limit of detection.^52^

In our model, the wall width of the tip is at the order of 1 nm, the nonlocal effect might come into appear, which cannot be effectively described by the simple hybridization scheme. Ciracì *et al.* studied the limits of plasmonic enhancement in a system containing a film-coupled Au nanosphere above a gold film. When the film thickness decreased to 1 nm, nonlocality effect caused the reduction of field enhancement and blueshift of the resonant wavelength compared to the local theoretical prediction.^53^ However, for a realistic value of the nonlocal parameter of 1.0 × 10^6^ m s^−1^, theoretical and experimental results indicated that the field enhancement and the resonant frequency are both change slightly, which means a slight reduction of sensitivity.

## 5. Conclusions

In this paper, we study a narrow Fano resonance of 3D nanocrescent and its application in single-molecular detection. The Fano resonance depends not only on the gap size but also on the height. The Fano resonance originates from the interference between the quadrupolar mode supported by the horizontal crescent and the dipolar mode supported by the nanotip oscillating along the height direction. By introducing a mathematical model based on the physical generation mechanisms of the Fano lineshape, we studied the physical mechanism responsible for the formation of the narrow Fano lineshape of the absorption spectra under a horizontal incident light. The narrow linewidth is due to strong resonant absorption because of the dipolar mode in the vertical direction overlapping perfectly with the quadrupolar mode in the horizontal direction, causing a narrow Fano resonance in the scattering spectra. When the height is *H* = 30 nm, the linewidth is as narrow as 10 nm.

The narrow Fano resonance is highly sensitive to a single nanoparticle trapped by the nanocrescent, which increases the overall figure of merit. The wavelength shift is 4.03 nm as a protein nanoparticle with radius of 2.5 nm is trapped by the nanocrescent. The wavelength shift increases linearly with the refractive index with the relation of Δ*λ* = 22.10*n* − 28.80, and increases with the size of trapped nanoparticle following a relation of Δ*λ* = 0.826 × *r*^1.672^. These provide a real-time label-free detection system for single nanoparticles in the future.

## Conflicts of interest

There are no conflicts to declare.

## Supplementary Material
